# Middle East Respiratory Syndrome Coronavirus, Saudi Arabia, 2017–2018

**DOI:** 10.3201/eid2511.190726

**Published:** 2019-11

**Authors:** Ahmed Hakawi, Erica Billig Rose, Holly M. Biggs, Xiaoyan Lu, Mutaz Mohammed, Osman Abdalla, Glen R. Abedi, Ali A. Alsharef, Aref Ali Alamri, Samar Ahmad Bereagesh, Kamel M. Al Dosari, Saad Abdullah Ashehri, Waad Ghassan Fakhouri, Saleh Zaid Alzaid, Stephen Lindstrom, Susan I. Gerber, Abdullah Asiri, Hani Jokhdar, John T. Watson

**Affiliations:** Ministry of Health, Riyadh, Saudi Arabia (A. Hakawi, M. Mohammed, O. Abdalla, A.A. Alsharef, A.A. Alamri, S.A. Bereagesh, K.M. Al Dosari, S.A. Ashehri, W.G. Fakhouri, S.Z. Alzaid, A. Asiri, H. Jokhdar);; Centers for Disease Control and Prevention, Atlanta, Georgia, USA (E.B. Rose, H.M. Biggs, X. Lu, G.R. Abedi, S. Lindstrom, S.I. Gerber, J.T. Watson)

**Keywords:** Middle East respiratory syndrome, MERS, MERS-CoV, coronavirus infections, respiratory viruses, viruses, Saudi Arabia

## Abstract

We characterized exposures and demographics of Middle East respiratory syndrome coronavirus cases reported to the Saudi Arabia Ministry of Health during July 1–October 31, 2017, and June 1–September 16, 2018. Molecular characterization of available specimens showed that circulating viruses during these periods continued to cluster within lineage 5.

Middle East respiratory syndrome (MERS) coronavirus (MERS-CoV) epidemiology in Saudi Arabia is characterized by healthcare-associated outbreaks (*1*,*2*), occasional household-contact transmission (*3*), and sporadic cases without apparent links to other known cases (*4*,*5*). Since 2015, healthcare-associated transmission has decreased as infection prevention and control practices have improved (*6*); however, sporadic cases continue to be reported, often associated with contact with dromedaries (*4*,*7*). Surveillance and routine investigation of recent MERS cases are critical to monitor the epidemiology of this emerging pathogen. We characterized exposures and demographics of MERS cases reported to the Saudi Arabia Ministry of Health during July 1–October 31, 2017, and June 1–September 16, 2018, and performed molecular characterization of available specimens to describe circulating viruses during these periods.

We summarized demographics and exposures using Ministry of Health investigation data. To further characterize exposures among sporadic cases (no known epidemiologic link to a hospital outbreak or known case) reported during July 1–October 31, 2017, we conducted telephone interviews using a standardized questionnaire addressing demographics and activities during the 14 days before symptom onset (exposure period). For deceased or unavailable patients, we interviewed proxies. We did not conduct interviews for cases reported during June 1–September 16, 2018; this period was selected because of specimen availability. Cases were confirmed by testing respiratory specimens with MERS-CoV real-time reverse transcription PCR assays (*8*). We shipped 20 specimens to the US Centers for Disease Control and Prevention for genome sequence analysis (*9*).

During July 1–October 31, 2017, a total of 61 MERS cases were reported from 12 of 13 administrative regions. Median patient age was 50 (range 10–89) years; 43 (70%) were male, and 23 (38%) died. Nine (15%) cases were associated with a hospital outbreak, 10 (16%) were household contacts of known cases, and 42 (69%) were classified as sporadic and further investigated. During November 2017, we interviewed 35 (83%) sporadic case-patients, 9 directly and 26 by proxy; 7 were unavailable. Among the 42 sporadic case-patients, median age was 57 (range 25–89) years; 35 (83%) were male, and 33 (79%) reported underlying conditions, most commonly diabetes (n = 24) and hypertension (n = 23). All were symptomatic and hospitalized; 22 (52%) died. During the exposure period, 21 (50%) sporadic case-patients reported camel contact: 12 had direct contact (touching), 5 indirect contact (visiting a setting with camels or exposure to others with direct camel contact), and 4 contact that could not be further classified. Among patients with camel contact, 6 also reported visiting a healthcare facility without a known MERS-CoV outbreak for a reason unrelated to their subsequent MERS illness. Of the 21 sporadic case-patients without camel contact, 8 (38%) visited a healthcare facility without a known MERS-CoV outbreak, 5 (24%) denied high-risk (camel- or healthcare-related) exposures, 1 (2%) was reclassified after interview as a contact of a previously identified case-patient, and 7 (17%) had insufficient exposure data to further characterize.

We analyzed MERS-CoV whole-genome sequences from 4 sporadic cases from 2017 (Figure). All sequences demonstrated 99.9% nucleotide (nt) sequence identity and clustered together within lineage 5, the predominant circulating lineage since 2015 (*9*). Three were from the same region and had an identical 3-nt in-frame deletion in open reading frame (ORF) 4b (Figure). Among case-patients with the deletion, 1 had incomplete exposure data, but 2 reported visiting the same hospital during their nonoverlapping exposure periods. None reported camel contact. The fourth patient did not report high-risk exposures. All 4 patients died; interviews for 3 were conducted via proxy.

**Figure:**
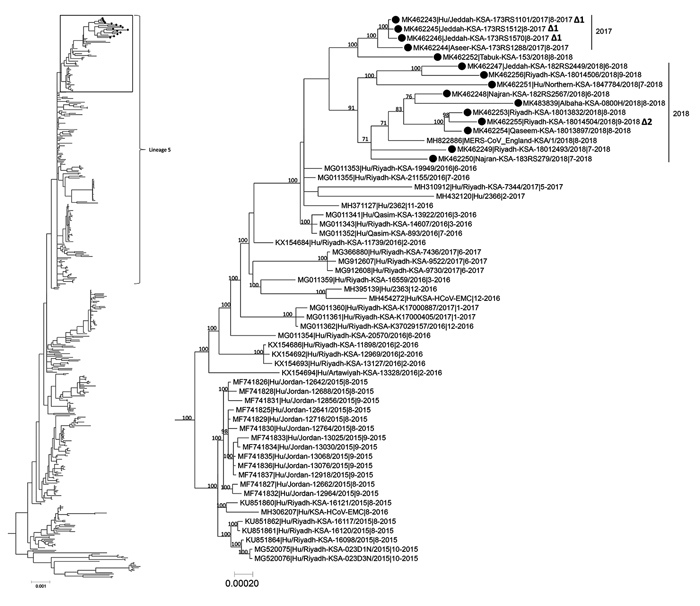
Phylogenetic tree of MERS-CoV whole-genome sequences obtained in Saudi Arabia (black dots) compared with 472 previously published human and camel genome sequences from GenBank. Tree inferred using MrBayes version 3.2.6 (https://nbisweden.github.io/MrBayes) under a general time-reversible model of nucleotide substitution with 4 categories of γ-distributed rate heterogeneity and a proportion of invariant sites. Box at the top of the tree on the left shows location of the tree on the right, showing lineage 5. Δ1 indicates 3-nt in-frame deletion in open reading frame 4a and Δ2 indicates 66-nt in-frame deletion in open reading frame 1a. Clade-credibility values >70% are indicated at selected nodes. Sequences from this study include GenBank accession numbers MK462243–MK462256 and MK483839. Scale bars indicate nucleotide substitutions per site. MERS-CoV, Middle East respiratory syndrome coronavirus; Hu, human; KSA, Kingdom of Saudi Arabia.

During June 1–September 16, 2018, a total of 32 MERS cases were reported from 10 administrative regions (*10*). Median patient age was 56 (range 29–84) years; 28 (88%) were male, and 23 (72%) reported underlying conditions, most commonly diabetes (n = 19) and hypertension (n = 15). Eleven (34%) patients died. Seven (22%) cases were in household contacts of known cases, 2 (6%) were healthcare-associated, and 23 (72%) were considered sporadic. Twelve (52%) sporadic case-patients reported camel contact. We obtained whole genome sequences from 11 specimens and full or partial spike gene sequences from 5. One virus sequence obtained in 2018 from a patient from the Tabuk region was most similar to those in the 2017 clade; the other 10 virus sequences showed 99.5%–99.9% nt identity and formed a separate clade. This clade included a sequence from a 2018 case-patient from Saudi Arabia who traveled to the United Kingdom. Sequence variability among the 2018 viruses supports the disparate geographic origin of these cases. A case-patient in the Riyadh region who reported no high-risk exposures had a 66-nt in-frame deletion in ORF1a (Figure; GenBank accession no. MK462255).

In conclusion, dromedary contact was common for recent case-patients with sporadic MERS-CoV in Saudi Arabia, indicating continued zoonotic transmission to humans. MERS-CoV infection continues to be reported periodically among those without known high-risk exposures, warranting further investigation. Recently circulating viruses remain in lineage 5, the predominant circulating strain in Saudi Arabia since 2015. Sporadic unique ORF sequence deletions continue to be observed, highlighting the importance of ongoing molecular virology surveillance.
